# Species distribution models for invasive Eurasian watermilfoil highlight the importance of data quality and limitations of discrimination accuracy metrics

**DOI:** 10.1002/ece3.8002

**Published:** 2021-08-13

**Authors:** Shyam M. Thomas, Michael R. Verhoeven, Jake R. Walsh, Daniel J. Larkin, Gretchen J. A. Hansen

**Affiliations:** ^1^ Department of Fisheries Wildlife & Conservation Biology and Minnesota Aquatic Invasive Species Research Center University of Minnesota St. Paul Minnesota USA; ^2^ Minnesota Department of Natural Resources St. Paul Minnesota USA

**Keywords:** abundance–suitability relationship, discrimination accuracy, functional accuracy, invasion risk, pseudoabsences, random forest, spatial autocovariate, water temperature

## Abstract

**Aim:**

Availability of uniformly collected presence, absence, and abundance data remains a key challenge in species distribution modeling (SDM). For invasive species, abundance and impacts are highly variable across landscapes, and quality occurrence and abundance data are critical for predicting locations at high risk for invasion and impacts, respectively. We leverage a large aquatic vegetation dataset comprising point‐level survey data that includes information on the invasive plant *Myriophyllum spicatum* (Eurasian watermilfoil) to: (a) develop SDMs to predict invasion and impact from environmental variables based on presence–absence, presence‐only, and abundance data, and (b) compare evaluation metrics based on functional and discrimination accuracy for presence–absence and presence‐only SDMs.

**Location:**

Minnesota, USA.

**Methods:**

Eurasian watermilfoil presence–absence and abundance information were gathered from 468 surveyed lakes, and 801 unsurveyed lakes were leveraged as pseudoabsences for presence‐only models. A Random Forest algorithm was used to model the distribution and abundance of Eurasian watermilfoil as a function of lake‐specific predictors, both with and without a spatial autocovariate. Occurrence‐based SDMs were evaluated using conventional discrimination accuracy metrics and functional accuracy metrics assessing correlation between predicted suitability and observed abundance.

**Results:**

Water temperature degree days and maximum lake depth were two leading predictors influencing both invasion risk and abundance, but they were relatively less important for predicting abundance than other water quality measures. Road density was a strong predictor of Eurasian watermilfoil invasion risk but not abundance. Model evaluations highlighted significant differences: Presence–absence models had high functional accuracy despite low discrimination accuracy, whereas presence‐only models showed the opposite pattern.

**Main conclusion:**

Complementing presence–absence data with abundance information offers a richer understanding of invasive Eurasian watermilfoil's ecological niche and enables evaluation of the model's functional accuracy. Conventional discrimination accuracy measures were misleading when models were developed using pseudoabsences. We thus caution against the overuse of presence‐only models and suggest directing more effort toward systematic monitoring programs that yield high‐quality data.

## Graphical abstract

Multiple niche models for invasive Eurasian watermilfoil were developed and compared to evaluate the importance of response data and model accuracy metrics. Abundance data are crucial in gaining a fuller understanding of EWM invasion and improve model evaluation metrics. Thus, the study cautions against the overuse of simple presence–absence niche models.

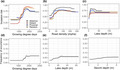

## 1 INTRODUCTION

Species distribution models (SDMs; *aka* ecological niche models) are among the most widely used modeling approaches in ecology and conservation science (Elith & Leathwick, 2009; Johnson & Gillingham, 2005). Rooted in ecological niche theory (Higgins et al., 2012; Pulliam, 2000; Soberón, 2007), the goal of species distribution models is to quantify species–environment correlations that best capture the underlying true (but unknown) relationship between environmental conditions and species habitat suitability. Most commonly, SDMs are used to predict the occurrence or abundance of species within and outside of their current ranges and/or under future environmental conditions, for example, species responses to future climate change, or the potential for invasive species to establish in new habitats (reviewed by Guisan & Thuiller 2005; Barbet‐Massin et al., 2018; Mikulyuk et al., 2020).

The predictive performance of SDMs varies depending on conceptual assumptions, methodological specifics, and ecological information used in developing SDMs (Hernandez et al., 2006; Merow et al., 2014; Synes & Osborne, 2011; Wiens et al., 2009). Efforts have been made to improve SDM performance and utility by increasing the quality of the training data used—specifically by including species abundance data instead of solely relying on presence and/or absence data (Howard et al., 2014; Kulhanek et al., 2011; Mi et al., 2017; Mikulyuk et al., 2020). Local abundance data indicate both habitat suitability and quality and can account for differences in microhabitat conditions driven by factors such as resource availability and biotic interactions (Boulangeat et al., 2012; Pearce & Ferrier, 2001; VanDerWal et al., 2009; Verhoeven et al. 2020; Weber et al., 2017). Thus, the inclusion of abundance data may improve predictive performance of occurrence‐based SDMs and also offer a richer understanding of species’ niches and ecological effects (Carrascal et al., 2015; Howard et al., 2014; Warren et al., 2020).

Habitat suitability predictions from SDMs are typically based on species occurrence (presence–absence) data and are essentially occurrence probability or habitat suitability estimates. However, SDMs often do not involve true absence information, as confirmed absences are typically unavailable in most survey and monitoring databases. Given this difficulty to obtain absence information, several presence‐only SDM approaches have been developed in which false absences (typically referred to as “pseudoabsences”) are used in place of true absences. These pseudoabsences are locations where the species has not been documented, but in fact may not have been surveyed, and therefore, the true status of the species is unknown. The use of pseudoabsences involves many assumptions and needs careful planning (Barbet‐Massin et al., 2012; Lobo & Tognelli, 2011; Senay et al., 2013), and it is not surprising that studies generally suggest using absence data whenever they are available (Brotons et al., 2004; Václavík & Meentemeyer, 2009).

Habitat suitability predicted from binary occurrence data can predict species abundance through the “abundance–suitability” relationship, that is, the correlation between predicted probability of occurrence and current (or potential) abundance (Nielsen et al., 2005; VanDerWal, Shoo, Johnson, et al., 2009). The abundance–suitability relationship is built on the assumption that locations that are more suitable for species establishment will also support higher abundances. However, the existence and strength of the abundance–suitability relationship can vary substantially, with recent studies showing only weak correlations (Baer & Maron, 2020; Dallas & Hastings, 2018; Mills, 2021; Weber et al., 2017). In a meta‐analysis by Weber et al. (2017), the strength of this correlation was found to vary depending on several factors, including the environmental variables used to predict suitability. For instance, SDMs built using coarse‐scale climatic variables result in relatively weak abundance–suitability correlations compared to SDMs that also include local microclimatic variables and/or biotic factors (Dallas & Hastings, 2018; Weber et al., 2017). Despite these uncertainties, the strength of the abundance–suitability relationship provides a meaningful evaluation metric for occurrence‐based SDMs (Lobo et al., 2008), which Warren et al. (2020) refer to as “functional accuracy.” Unlike “discrimination accuracy” measures such as AUC (area under the receiver operating characteristic curve; Fielding & Bell, 1997), functional accuracy measures based on abundance–suitability correlation strength have clear biological relevance that can be leveraged for empirical applications (Warren et al., 2020). Moreover, functional accuracy metrics avoid known problems with discrimination accuracy measures, as latter can be particularly troublesome when models include pseudoabsences from unsampled background distributions (Jiménez‐Valverde, 2012; Lobo et al., 2008).

The abundance of an invasive species has long been considered one of the key components that determine its potential impact (Latzka et al., 2016; Parker et al., 1999; Thomsen et al., 2011; Yokomizo et al. 2009). At the same time, there is considerable spatial heterogeneity in abundance across invasive species’ distributions, with relatively few locations typically supporting high abundance (Hansen et al., 2013). Given the ecological significance and inherent spatial variability of abundance, SDMs of invasive species increasingly combine occurrence and abundance data to predict invasion risk and impact, respectively (Bradley, 2013; Januario et al., 2015; Kulhanek et al., 2011; Mikulyuk et al., 2020). Such approaches have highlighted discontinuities between predicted locations of invasion risk and invasion impact (Bradley, 2016; Mikulyuk et al., 2020; Thomas et al., 2017). In short, it is now well understood that the availability of high‐quality data, especially absences and relative abundance measures, is crucial for developing ecologically accurate SDMs (Bradley et al. 2018).

The importance of data type and quality in SDM applications is widely acknowledged (e.g., Bradley et al. 2018; Guillera‐Arroita et al., 2015; Howard et al., 2014; Leroy et al., 2018). However, few studies to our knowledge have simultaneously addressed the roles of absence, pseudoabsence, and abundance information (Aarts et al., 2012; Carrascal et al., 2015). While studies have exploited abundance data to evaluate the functional utility of SDMs as measured by abundance–suitability correlation strength (reviewed by Weber et al., 2017), it remains unclear how pseudoabsences (in presence‐only SDMs) affect the abundance–suitability relationship (but see Warren et al. (2020) for a simulation‐based assessment). In the only known study by Carrascal et al. (2015), presence–absence models were found to be superior to pseudoabsence‐based models in predicting local and regional abundance. However, the study by Carrascal does not probe the role of different pseudoabsence selection strategies in affecting functional accuracy.

The inclusion of spatial lag term (such as an autocovariate) is yet another common technique employed in SDMs to account for spatial autocorrelation in environmental drivers and/or species distribution data (Dormann et al., 2007). Addressing spatial autocorrelation is particularly important for invasive SDMs since biological invasions are often contagious processes constrained by dispersal limitation and proximity to anthropogenic disturbances (Václavík et al., 2012). Moreover, the incorporation of spatial lag terms is known to improve model performance measures such as AUC (and other discrimination accuracy measures) and estimation of species–environment relationship (Crase et al., 2012; Václavík et al., 2012). Yet again, it remains unknown if the inclusion of a spatial autocovariate will also improve functional accuracy.

Here, we make use of a rich long‐term aquatic plant monitoring dataset to build SDMs based on systematically collected presence, absence, and abundance data. We develop multiple SDMs for the invasive plant Eurasian watermilfoil (*Myriophyllum spicatum* L.; hereafter EWM) using response variables that differ in quality and definition. The goals of our study are to gain a comprehensive understanding of the environmental drivers of EWM invasion and simultaneously explore how data quality influences modeling results and interpretations. Specifically, we (a) develop multiple SDMs trained with EWM presence–absence, presence‐only, and abundance datasets with a focus on determining the relative importance of drivers for EWM occurrence versus abundance; (b) explore the effects of pseudoabsences and spatial lag terms on model results and performance; and (c) evaluate and compare presence–absence and presence‐only models based on discrimination accuracy and functional accuracy.

## 2 METHODS

### 2.1 Study area and species

**FIGURE 1:**
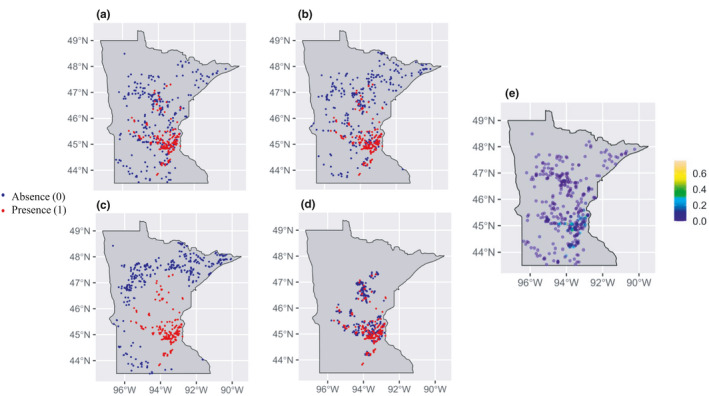
Maps of study area showing invasive EWM distribution and abundance patterns used to construct (a) presence–absence models, (b) presence‐only models with random pseudoabsences, (c) presence‐only models with distant pseudoabsences, (d) presence‐only models with proximal pseudoabsences, and (e) abundance (frequency of occurrence) models. Absences and pseudoabsences are shown as solid blue‐colored dots, whereas presences are shown as solid red colored dots. Abundance is measured as frequency of occurrence with values ranging from 0 (complete absence of EWM) to 1 (all sampled points occupied)

Our study focuses on the distribution and abundance of invasive EWM across the lake‐rich landscape of Minnesota, USA (Figure 1). EWM is a submerged aquatic perennial plant native to Europe and Asia that was likely introduced to North America in the late 19th century (Nichols & Shaw, 1986). In Minnesota, the earliest known EWM occurrences were recorded in 1985 (Smith & Barko, 1990) and EWM is currently documented in more than 300 lakes (https://www.dnr.state.mn.us/invasives/ais/infested.html). Among the characteristics that make EWM a successful invader are its efficient dispersal mechanisms and rapid growth early in the growing season (Grace & Wetzel, 1978; Smith & Barko, 1990). Under optimal conditions, EWM can attain high densities and form dense mats on the water's surface, which can negatively affect aquatic ecosystems by reducing species richness and altering water quality, food web interactions, and underwater habitat structure (Boylen et al., 1999; Cheruvelil et al., 2001; Madsen et al., 1991; Webb et al., 2016). EWM infestation is also associated with poor esthetic appeal and lower economic value of lakeshore properties (Goodenberger & Klaiber, 2016; Zhang & Boyle, 2010).

### 2.2 EWM response data

Lake‐specific EWM occurrence and abundance data were obtained from point‐intercept surveys of aquatic plants in lakes across Minnesota (Verhoeven, Glisson, et al., 2020; Verhoeven et al., 2021). Between 1995 and 2019, 1,534 Minnesota lakes were surveyed at least once, and 365 lakes were found to have EWM. All surveys were conducted via the rake‐based point‐intercept methodology, which entails sampling macrophytes (aquatic plants and macroalgae) from a boat using a metal rake dragged along the bottom at a predefined grid of sample locations within lakes’ photic/littoral zones (Hauxwell et al., 2010; Madsen & Wersal, 2017; Perleberg et al., 2016). This method provides occurrence data at the lake level and occurrence data at the point level.

We characterized EWM occurrence using two broad approaches: presence–absence data and presence‐only data. The presence–absence approach relies exclusively on surveyed lakes, which provides true absences (i.e., lakes that were surveyed but no EWM was found). The presence‐only approach encompasses EWM‐invaded lakes together with unsurveyed lakes not known to have EWM, which are treated as pseudoabsences. Using three different spatial draws of pseudoabsences (i.e., unsurveyed lakes), the presence‐only data were further categorized into (a) random presence‐only data, where pseudoabsences were drawn randomly from the study area; (b) distant presence‐only data, where pseudoabsences were drawn from unsurveyed lakes outside a convex hull surrounding invaded lakes; and (c) proximal presence‐only data, with pseudoabsences drawn from unsurveyed lakes contained within the invaded‐lakes convex hull. These three different presence‐only datasets represent the commonly used pseudoabsence selection strategies (Senay et al., 2013) and provide a range of scenarios for comprehensive comparison and evaluation of model predictions.

EWM abundance for a given lake was calculated as the proportion of surveyed point locations within a lake with positive EWM detection (Mikulyuk et al. 2020; Verhoeven et al., 2020). Lake‐level EWM abundance is thus a measure of the proportion of sampled points within a lake in which EWM was found, hereafter the “frequency of occurrence.” Unlike the binary presence–absence and presence‐only response variable, frequency of occurrence is a measure of plant cover within the lake that can range from 0 to 1 (i.e., from complete absence to detection at 100% of sampled points within a lake).

### 2.3 Environmental predictors

We collated 11 lake‐specific environmental predictors from multiple sources that provide a snapshot of lake physical and chemical characteristics and surrounding landscape conditions (Table 1). Our choice of variables was primarily based on previous published works on EWM invasion (Buchan & Padilla, 2000; Roley & Newmann, 2008; Tamayo & Olden, 2014). Lake morphometric variables included lake size and maximum depth and were sourced from the Minnesota Department of Natural Resources (MNDNR) hydrological shape file (https://gisdata.mn.gov/dataset/water‐dnr‐hydrography). EWM spring growth is initiated when water temperature exceeds 10°C (Stanley & Naylor, 1972) and growth is fastest at relatively high temperatures (30–35°C) (Smith & Barko, 1990). To account for the dependence of EWM on water temperature, we used lake‐specific growing degree days with a base temperature of 10°C, calculated from simulated water temperatures (Winslow et al., 2017). Lake‐level growing degree days were estimated as averages of water surface temperatures between the years 1995 and 2015. Lake water chemistry variables included pH, conductance, chlorophyll‐*a*, and total phosphorus, all of which were gathered from annual lake monitoring surveys conducted by the Minnesota Pollution Control Agency (MPCA). Water chemistry measures for a lake were recorded as the average measure of all sampling events within a lake between the years 1995 and 2019. Lake connectivity variables comprised road density and stream density measures within 500 m from lake edges, which were sourced from the LAGOS database (Soranno et al., 2017). Water clarity was indexed by Secchi depth compiled from the MPCA and MNDNR databases for all years between 1995 and 2018. If data were available for sub‐basins of multibasin lakes, we kept the data at the sub‐basin scale whenever possible. If multiple Secchi observations were recorded on the same date within a lake or sub‐basin, we used the daily median. The daily median values were then averaged across years to provide lake‐specific mean Secchi depth measures. Water color was indexed by remotely sensed CDOM a_440_ (colored dissolved organic matter absorption coefficients at 440 nm, averaged over years 2015–2016) from Olmanson et al. (2020).

**TABLE 1:** Summary statistics of the 11 lake‐level predictor variables used in the Random Forest SDMs to predict Eurasian watermilfoil invasion risk and frequency of occurrence. Longitude and latitude summarize the geographic distribution of the sampled lakes

Variable name (units)	Mean	*SD*	Min	Max
CDOM_a440 (m^−1^)	2.38	2.27	0	15.1
Chlorophyll‐A (µg/L)	31	84	0.093	2,231
Conductance (µS/cm)	313	215	8.53	1,750
Water temperature degree days (base 10, °C*days)	1671	225	1,078	2,465
Lake depth (m)	10.2	9.58	0.914	141
Lake size (acres)	759	4984	8.82	128,251
pH	8.03	0.633	6.02	9.71
Phosphorus (mg/L)	0.090	0.116	0.004	1.18
Road density (m/ha)	40.6	29.3	0	211
Secchi depth (m)	2.35	1.48	0.167	12.9
Stream density (m/ha)	4.18	3.5	0	21.2
Latitude (DD)	46.1	1.22	43.5	48.5
Longitude (DD)	−93.9	1.21	−96.7	−90.1

The final dataset included only the lakes for which all environmental variables were available, with 184 invaded and 284 uninvaded lakes forming the presence–absence data (Figure 1a), and an additional 801 unsurveyed lakes leveraged as pseudoabsences in presence‐only datasets (Figure 1b–d). In total, 1,269 lakes with complete information on all lake‐level predictors form the crux of all our models and analyses. These lakes span a broad climatic range, from 43.5°N to 48.5°N, and capture a wide variety of aquatic habitats and surrounding landscape conditions (Table 1). In the random, distant, and proximal presence‐only datasets, 284 pseudoabsences were strategically selected from the 801 unsurveyed lakes to replace the true absences (Figure 1b–d; see previous section for details on pseudoabsence selection strategies). Finally, the frequency of occurrence of EWM for the 184 invaded lakes, together with the 284 uninvaded lakes, formed the EWM abundance dataset (Figure 1e). EWM frequency of occurrence values ranged from 0 to 0.8, and the distribution was strongly right skewed, with few lakes having high EWM frequency of occurrence.

### 2.4 Random Forest SDMs

We used the Random Forest (RF) algorithm (“randomForest” package in R; Liaw & Wiener, 2002) to model the distribution and abundance of EWM invasion in lakes. RF models combine the strength of multiple classification trees with a bagging approach (since they combine predictions from multiple decision trees) to make accurate predictions that are resistant to overfitting while also allowing for nonlinear response curves (Cutler et al., 2007; Evans et al., 2011). Another advantage of using RF models is the ability to directly estimate relative variable importance, which allows an identification of influential ecological predictors (Cutler et al., 2007). Overall, RF is considered to have high performance accuracy and stability (Duan et al., 2014).

We developed five different categories of RF models, one for each individual response type: presence–absence model, presence‐only model with random pseudoabsences, presence‐only with distant pseudoabsences, presence‐only with proximal pseudoabsences, and an abundance model. Each RF model included the 11 environmental variables described previously. An additional set of five RF models were developed with all the environmental predictors plus an auxiliary spatial lag term to account for spatial autocorrelation. The spatial lag term was incorporated in the form of an inverse distance‐weighted autocovariate using the “spdep” package (Bivand et al., 2013). Prior to incorporating the spatial autocovariate, assessment of spatial autocorrelation using Moran's I showed significant and positive spatial autocorrelation in the residuals of both presence–absence and abundance models; this was especially evident in the case of presence–absence model residuals (Moran's I for presence–absence model residuals = 0.047, *p* < 0.0001; for abundance model residuals = 0.012, *p* = 0.04). All model runs involved splitting the datasets into 70 percent training data and 30 percent test data for estimating model accuracy. Overall, the 10 distinct RF models provide a detailed comparison of EWM’s predicted niche in relation to multiple types of distribution measures, with and without accounting for spatial autocorrelation. We provide a detailed overview of all the steps involved in the RF species distribution modeling based on the ODMAP template developed recently (Zurell et al., 2020; see Table S1 for ODMAP).

### 2.5 Model comparisons and evaluation

One of the main goals of our study was to compare the results of presence–absence, presence‐only, and abundance‐based RF models with a focus on understanding how the effects of lake‐specific factors on EWM invasion risk and abundance vary across the different models. To do so, we first compared relative importance of predictor variables across all models. For both occurrence‐based (i.e., presence–absence and presence‐only) and abundance models, relative importance of variables was calculated as percent change in mean‐squared error (*MSE*) score when a variable was permuted (Liaw & Wiener, 2002). Next, the response curves of key predictors were compared across all models using partial dependency plots. Additionally, we assessed change in relative importance of variables and response curves for all models after including the spatial autocovariate.

We were also interested in comparing discrimination accuracy and functional accuracy of presence–absence and presence‐only models. Three conventional discrimination accuracy statistics—AUC (area under the receiver operating characteristic curve), Kappa, and TSS (true skill statistic)—were calculated (see Allouche et al., 2006) using three different model evaluation methods: nonindependent, quasi‐independent, and spatially blocked. Kappa and TSS are threshold‐dependent measures, meaning that presence is assigned when probability of presence exceeds a threshold value. Threshold probability values were determined using the sensitivity–specificity equality approach, which is the preferred approach when it comes to ensuring both presence and absence have equal chances of being predicted correctly (Fielding & Bell, 1997; Liu et al., 2005). In the nonindependent evaluation approach, random 5‐fold cross‐validation with 50 iterations was executed, and the final discrimination accuracy statistics reported are the average of all iterations. In the quasi‐independent evaluation, discrimination accuracy statistics were the evaluation results from a single randomly assigned test dataset. In other words, the reported discrimination accuracy statistics in the quasi‐independent evaluation scenario are based on a single run of fixed training and test datasets. In the spatially blocked cross‐validation approach, the training and test data were derived from large contiguous spatial blocks using the “blockCV” package (Valavi et al., 2019). The size of the spatial block was set to 10 km for all occurrence models (except the presence‐only model with proximal pseudoabsences); this is approximately the largest scale at which lakes continued to show spatial clustering. For presence‐only models with proximal pseudoabsences, the size of the spatial block was set to 5 km to account for the restricted distribution of pseudoabsences. Spatial clustering of lakes was estimated using the pair correlation function, a point‐pattern analysis technique where clustering is assessed across multiple distances in a noncumulative manner by only counting points (lakes) that fall along discrete distance intervals (Baddeley et al.,^2015^).

Functional accuracy for each occurrence‐based model was evaluated by quantifying the strength of the abundance–suitability relationship between EWM frequency of occurrence and predicted suitability for EWM invasion. For each occurrence‐based model, the Pearson and Spearman correlation coefficient estimate of the relationship between predictions of the model and frequency of occurrence was considered as the measure of its functional accuracy. Hence, high positive values of the correlation coefficient imply predicted EWM invasion risk was a strong predictor of EWM frequency of occurrence. Studies have generally shown that inclusion of zeros can potentially inflate correlation coefficient estimates as it entails predictions of low suitability for zero abundance values (Dallas & Hastings, 2018). Given this sensitivity to zero abundance values, two separate estimates of Pearson's and Spearman's correlation coefficient (Pearson's *r* and Spearman's *⍴*) were reported, one that included zero values for frequency of occurrence (*r_all_* and *⍴_all_*) and another that excluded zero values (*r_nonzero_* and *⍴_nonzero_*). Finally, linear quantile regression models were used to further explore the abundance–suitability relationship. For both SDMs, with and without spatial autocovariate quantile regressions were used to examine the relationship between predicted EWM invasion risk and upper limits of EWM frequency of occurrence (i.e., the 50th, 75th, and 90th percentiles).

## 3 RESULTS

### 3.1 Model comparisons

**FIGURE 2:**
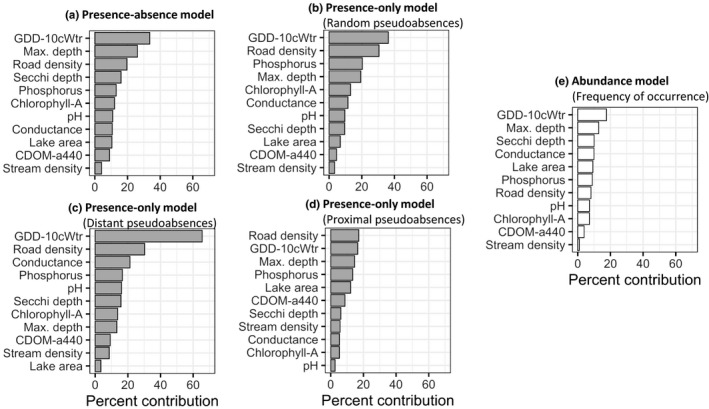
Percent relative contribution of predictors in EWM (a) presence–absence models, (b) presence‐only models with random pseudoabsences, (c) presence‐only models with distant pseudoabsences, (d) presence‐only models with proximal pseudoabsences, and (e) abundance (frequency of occurrence) models. Abbreviations: GDD‐10cWater = water temperature degree days (base 10), CDOM‐a440 = colored dissolved organic matter (absorbance at 440 nm)

Comparison of RF models of EWM presence–absence, presence‐only, and abundance revealed key differences and similarities. In terms of relative variable importance (Figure 2), growing degree days from modeled surface water temperature and lake depth were consistently among the two leading drivers across all models. Interestingly, road density around lakes was a key predictor of EWM occurrence but not abundance. Another notable difference between occurrence models (except for presence‐only models with proximal pseudoabsences) and the abundance model was the disproportionate influence of three variables on EWM occurrence—growing degree days, lake depth, and road density—compared to the relatively even contributions of nearly all variables in predicting EWM abundance. In addition, presence‐only models with distant pseudoabsences showed an exaggerated influence of growing degree days compared to other predictors. Among the three presence‐only models, relative variable importance of the presence‐only model with random pseudoabsences was most like that of the presence–absence model.

**FIGURE 3:**
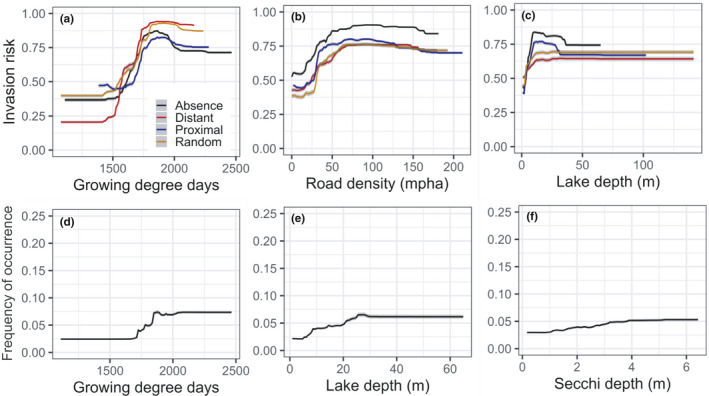
Partial dependence plots of RF models showing the predicted effects of the top three predictors on (a–c) EWM invasion risk and (d–f) frequency of occurrence. Line colors in the top panel highlight the different occurrence models, with the black colored lines depicting presence–absence models. The *y*‐axis of the bottom panel is reduced to show the frequency of occurrence response curves with clarity

**FIGURE 4:**
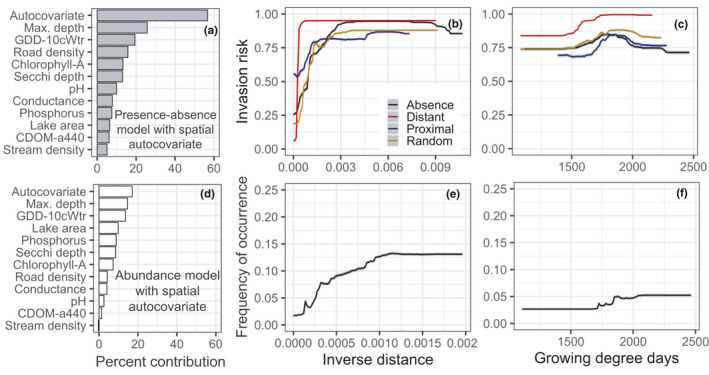
Plots showing the effect of including a spatial autocovariate in RF models of invasion risk and frequency of occurrence. Left panel shows the relative importance of predictors after including the autocovariate in (a) presence–absence models of EWM invasion risk and (d) models of EWM frequency of occurrence. Middle and right panels show partial response curves of the autocovariate on EWM (b) invasion risk and (e) frequency of occurrence and the subsequent effect on response curves of growing degree days (c & f)

Species–environment response curves of the three most important predictors further highlight subtle, yet important, differences among the models. Response curves of abundance models generally showed only modest changes in frequency of occurrence across a range of environmental variation, compared to occurrence models that exhibited strong shifts in invasion risk across the same range of environmental variation (Figure 3). Both EWM invasion risk and frequency of occurrence increased with increasing water temperature and plateaued at approximately 1,750°C*days (growing degree days); this was most apparent for occurrence models (Figure 3). Likewise, invasion risk and frequency of occurrence showed gradual increases with increasing lake maximum depth, up to ~30 m. Increase in road density around lakes increased only invasion risk, an effect that diminished at road densities greater than ~75 m/ha (Figure 3). Secchi depth was the third most important predictor in the abundance model, with clearer lakes (≥4‐m Secchi depth) more likely to support EWM occurrence. Notably, across the entire range of observed road density and depth values, presence–absence models predicted greater invasion risk compared to all pseudoabsence models, with clear differences in peak invasion risk estimates. Response curves of occurrence models showed overall similar patterns of invasion risk but with one notable difference: The response curves of all pseudoabsence‐based presence‐only models diverged considerably from the response curve of models with absence information. Inclusion of a spatial lag term strongly influenced the results; the spatial autocovariate was the leading predictor in all models in which it was included, with pronounced effects on both EWM invasion risk and frequency of occurrence (Figure 4). Concurrently, the inclusion of the spatial autocovariate led to growing degree days dropping in rank and its response curve showing a subtler impact on EWM invasion risk and frequency of occurrence.

### 3.2 Model evaluations

Regardless of the evaluation approach, discrimination accuracy statistics (AUC, TSS, and Kappa) revealed a similar pattern, with presence‐only models with random pseudoabsences and presence‐only models with distant pseudoabsences always being better predictors of EWM invasion than presence–absence models (Table 2). More specifically, discrimination accuracy measures of all occurrence models showed the following overall ranking: presence‐only models with distant pseudoabsences > presence‐only with random pseudoabsences > presence–absence > presence‐only with proximal pseudoabsences. However, functional accuracy measured as Pearson's correlation coefficients (*r_all_* and *r_nonzero_*) and Spearman's correlation coefficients (*⍴_all_* and *⍴_nonzero_*) showed a contrasting pattern, with presence–absence models recording the highest values compared to all other models. Overall, functional accuracy values showed the following ranking: presence–absence models > presence‐only with random pseudoabsences > presence‐only with proximal pseudoabsences > presence‐only with distant pseudoabsences. Inclusion of a spatial autocovariate generally improved discrimination accuracy and functional accuracy values with some exceptions. Specifically, inclusion of a spatial autocovariate reduced discrimination accuracy estimates under the quasi‐independent approach for presence‐only models with distant and proximal pseudoabsences, and it also reduced Pearson's *r* for presence‐only models with distant pseudoabsences. Averaging all the discrimination accuracy estimates provides a clearer picture, with only presence‐only models with proximal pseudoabsences continuing to show a slightly lower Kappa estimate when an autocovariate is included.

**TABLE 2:** Summary of discrimination accuracy (AUC, TSS, and Kappa) and functional accuracy metrics calculated for all EWM occurrence SDMs with and without a spatial autocovariate. Numbers with a downward arrow subscript indicate drop in discrimination accuracy and functional accuracy estimates after including spatial autocovariate. Functional accuracy shown here is calculated as the Pearson correlation coefficient (*r*_all_ and *r*_nonzero_) and Spearman correlation coefficient (*⍴*_all_ and *⍴*_nonzero_) of EWM abundance–suitability relationship (*see Methods for details*)

Model type	Discrimination accuracy												Functional accuracy			
	Nonindependent			Quasi‐independent			Spatially blocked			Combined average			Abundance–suitability correlation			
	AUC	TSS	Kappa	AUC	TSS	Kappa	AUC	TSS	Kappa	Avg. AUC	Avg. TSS	Avg. Kappa	*r_all_*	*r_nonzero_*	*⍴_all_*	*⍴_nonzero_*
**Presence–absence**	0.83	0.56	0.54	0.82	0.44	0.43	0.76	0.43	0.44	**0.80**	**0.48**	**0.47**	**0.58**	**0.39**	**0.68**	**0.40**
**Presence–absence + Autocovariate**	0.88	0.65	0.63	0.90	0.72	0.70	0.86	0.54	0.53	**0.88**	**0.69**	**0.62**	**0.59**	**0.40**	**0.69**	**0.43**
**Presence‐only** (random pseudoabsence)	0.88	0.66	0.65	0.87	0.63	0.62	0.86	0.54	0.54	**0.87**	**0.61**	**0.60**	**0.47**	**0.28**	**0.62**	**0.26**
**Presence‐only** (random pseudoabsence) + **Autocovariate**	0.91	0.69	0.67	0.86_↓_	0.58_↓_	0.57_↓_	0.89	0.59	0.62	**0.89**	**0.62**	**0.62**	**0.51**	**0.36**	**0.65**	**0.33**
**Presence‐only** (distant pseudoabsence)	0.98	0.87	0.86	0.87	0.61	0.59	0.91	0.73	0.73	**0.92**	**0.74**	**0.73**	**0.40**	**0.26**	**0.61**	**0.20**
**Presence‐only** (distant pseudoabsence) + **Autocovariate**	0.99	0.99	0.99	1	1	1	0.99	0.98	0.99	**0.99**	**0.99**	**0.99**	**0.26_↓_**	**0.24_↓_**	**0.62**	**0.21**
**Presence‐only** (proximal pseudoabsence)	0.76	0.43	0.43	0.86	0.62	0.61	0.64	0.32	0.35	**0.75**	**0.45**	**0.46**	**0.48**	**0.25**	**0.64**	**0.30**
**Presence‐only** (proximal pseudoabsence) + **Autocovariate**	0.79	0.48	0.47	0.81_↓_	0.53_↓_	0.52_↓_	0.76	0.34	0.36	**0.79**	**0.45**	**0.45_↓_**	**0.51**	**0.36**	**0.65**	**0.41**

The final estimates of average discrimination accuracy measures and correlation derived functional accuracy measures are shown in bold.

**FIGURE 5:**
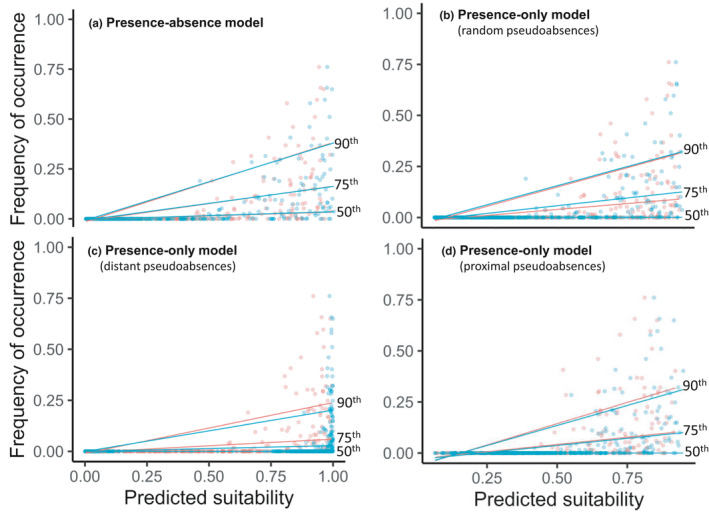
Plots showing EWM abundance–suitability relationship where the predicted suitability is based on presence–absence and three distinct presence‐only datasets. Red‐ and blue‐colored dots indicate models with and without the spatial autocovariate, respectively, with the corresponding quantile regression lines shown at the 50th, 75th, and 90th percentiles. Pearson correlation coefficients (functional accuracy measures) associated with these plots are shown in Table 2. Quantile regression coefficients associated with the regression lines are reported in Table S2

Abundance–suitability plots of EWM frequency of occurrence and predicted suitability for EWM invasion showed a wedge‐shaped relationship that varied among models with and without the spatial autocovariate (Figure 5a–d). Analysis of this wedge‐shaped relationship using quantile regression highlighted a strong positive relationship between probability of presence and frequency of occurrence, especially at the higher quantile levels (i.e., the 75th and 90th quantiles in Figure 5). However, there were noticeable differences in the quantile slope coefficients among the different SDMs, and the difference between 50th and 90th quantile slopes—a measure of strength of the wedge‐shaped relationship (Carrascal et al., 2015; Jiménez‐Valverde et al., 2021)—was greatest for presence–absence models and presence‐only models with proximal pseudoabsence (Table S2).

## 4 DISCUSSION

### 4.1 Drivers of EWM invasion risk and frequency of occurrence

We developed multiple SDMs to gain a deeper understanding of the ecological drivers of EWM invasion, while also evaluating the importance of data quality and model assumptions. Results of the presence–absence, presence‐only, and abundance‐based SDMs showed considerable overlap; however, there were key differences with important implications for model interpretation. For instance, while growing degree days and lake depth consistently predicted EWM occurrence and abundance, road density around lakes mattered most for occurrence models of invasion risk. Similar effects of surrounding landscape conditions on EWM invasion risk have been reported previously, especially with respect to boating, vehicular traffic, and lake visitation rates, all of which facilitate overland spread of EWM (Buchan & Padila, 2000; Kanankege et al., 2018; Mikulyuk et al., 2020). Moreover, while EWM invasion risk was overwhelmingly influenced by two key broad‐scale drivers—surface water temperature and road density—EWM frequency of occurrence was more evenly mediated by multiple factors, including water chemistry and clarity. Similar differences between predictors of EWM occurrence and abundance were reported in a recent study on EWM invasion in Wisconsin (Mikulyuk et al. 2020). This distinction between drivers of EWM occurrence and abundance suggests that efforts aimed at spread prevention versus management of established infestations might benefit from distinct prioritization strategies.

Temperature measures such as growing degree days can reflect species range limits related to growth and physiological processes. Appreciable levels of photosynthesis can occur in EWM at 10°C (Stanley & Naylor, 1972), and spring temperatures above 15°C are known to strongly influence both growth from overwintering roots and seed germination (Smith & Barko, 1990; Xiao et al., 2010). At the upper end of the temperature range limit, EWM can reportedly thrive in water temperatures as high as 35°C (Smith & Barko, 1990) and warmer temperatures can boost its photosynthetic activity (Grace & Wetzel, 1978). Hence, it is not surprising to find the strong influence of growing degree days in all models (Figure 2a–e), with both invasion risk and frequency of occurrence increasing markedly with increasing degree days (Figure 3a, d). Studies of EWM distribution and abundance have mostly ignored temperature as a predictor, with some exceptions (e.g., Mikulyuk et al., 2020, where maximum air temperature was found to be a strong driver of EWM invasion in Wisconsin lakes). It is therefore reassuring that growing degree days derived from water temperature had comparable effects on EWM invasion risk. It is worth noting that the steep increase in EWM invasion risk in response to growing degree days is partly attributable to a strong spatial gradient in temperature. This was evident when addition of the spatial autocovariate in the RF models decreased the relative importance and effects of growing degree days on EWM invasion risk and abundance (Figure 4). Moreover, the observed improvement in discrimination and functional accuracy measures when a spatial autocovariate was included suggests proximity to invaded lakes does matter, which can be attributed to latent, that is, unmeasured, spatially structured environmental drivers (such as water flow) and biotic factors (such as dispersal).

Across all RF models, lake depth was among the top three predictors of EWM occurrence and an even stronger predictor of EWM abundance (Figure 2). Lake depth has repeatedly been identified as a key driver of EWM invasion (Buchan & Padilla, 2000; Roley & Newman, 2008), wherein the probability of EWM invading a lake generally increases with depth and reaches an asymptote near a maximum depth of 10 m. This positive influence of lake depth is perhaps the consequence of variation in light availability wherein deeper lakes with clearer water not only provide suitable conditions for EWM to establish (Roley & Newman, 2008), but more importantly, such lakes are also more desirable for boating and fishing, which can lead to increased human activity that can elevate the likelihood of EWM introductions (Keeler et al., 2015). In this context, it is worth noting that Secchi depth was an important predictor of EWM abundance along with maximum depth (Figure 2e), suggesting lake depth and light availability together reflect within‐lake habitat availability and suitability. These findings are supported by recent work characterizing the species–environment associations of EWM from a microhabitat (within‐lake) niche perspective. Verhoeven, Glisson, et al. (2020) showed that depth, light, and growing degree days influenced not only habitat suitability for EWM, but also for other species likely to interact with EWM. Thus, the influence of depth could partially be a reflection of biotic interactions. Future work should further develop our understanding of how EWM abundance is influenced by biotic interactions, and how these interactions, as well as environmental associations, vary across scales.

Previous studies have reported pH and phosphorus as additional water chemistry influents of EWM invasion (Madsen, 1998; Buchan & Padilla, 2000; Roley & Newman, 2008). Interestingly, pH was always among the lowest‐ranked water chemistry variable across all models, except for presence‐only with distant pseudoabsences. As noted by Roley and Newman (2008), lakes in metropolitan Minnesota have relatively higher pH compared to its distant counterparts in more northern parts of the state. Moreover, lakes in northern and northeastern parts of Minnesota are mostly within a forested landscape and have colder water temperature, which together can also result in lower lake pH levels (Dunford et al., 2012). In short, the large climatic gradient and differences in surrounding landscape conditions might explain why pH is a better predictor of models with distant pseudoabsences. Thus, while EWM is known to occur in lakes with a wide range of pH levels, hard‐water lakes with moderately high pH levels tend to increase the likelihood of EWM occurrences. Phosphorus was a better predictor of EWM frequency of occurrence than of invasion risk, likely because elevated phosphorus stimulates EWM growth (Madsen, 1998).

### 4.2 Effects of pseudoabsence in presence‐only models

Presence‐only models with random, distant, and proximal pseudoabsences differed in significant ways from presence–absence models. In general, all three presence‐only models were able to discern the two key large‐scale drivers of EWM occurrence—growing degree days and road density. However, the relative importance of these two predictors varied depending on the pseudoabsence selection strategy. Distant pseudoabsences showed overwhelming influence of growing degree days compared to other variables, indicating that, unsurprisingly, water temperatures of invaded and uninvaded lakes became more disparate as pseudoabsences and invaded lakes were further apart. Conversely, the nearly equal effect of most variables in the proximal pseudoabsence models suggests nearby lakes share similar characteristics, and no single predictor alone can differentiate invaded and uninvaded lakes. Studies examining the effects of varying spatial extent of pseudoabsence locations have shown similar effects on relative variable importance, with models becoming simpler and dominated by one or two predictor variables with increasing distance from presence locations (Stokland et al., 2011; VanDerWal et al., 2009). Relative variable importance for the randomly selected pseudoabsences was closest to that of the presence–absence models. Random selection of pseudoabsences has often been the recommended approach as it samples a wide range of lakes across the study area (Barbet‐Massin et al., 2012; Wisz & Guissan, 2009). It was also apparent from the response curves that, despite overall similarity, presence‐only models showed clear discrepancies, with marginal effects on invasion risk either being under‐ or overpredicted. Pseudoabsences tend to distort species response curves and the degree of distortion depends on the pseudoabsence selection approach (Chapman et al., 2019; VanDerWal, Shoo, Graham, et al., 2009). In short, none of the presence‐only models were able capture the “true” EWM‐environment relationship as characterized by the presence–absence model. This perhaps also explains why in a previous study by Carrascal et al. (2015), MaxEnt models based on presence‐only data were inferior to presence–absence models in predicting abundance.

### 4.3 Limitations of discrimination accuracy metrics

Model discrimination and functional accuracy measures showed contrasting outcomes between presence–absence and presence‐only models that further underscore the inability of presence‐only models to capture species–environment relationships. The higher functional accuracy of presence–absence models compared to presence‐only models implies that ecologically relevant indicators of habitat suitability, such as EWM abundance, are best predicted by models that incorporate EWM absence information. The lower functional accuracy associated with presence‐only models also indicates the limitations of replacing EWM absence information with pseudoabsences. Notably, EWM presence‐only models often had higher discrimination accuracy measures, despite lower functional accuracy estimates, compared to presence–absence models. In other words, models with pseudoabsences had high discrimination capacity despite being poor predictors of EWM abundance. This ambiguous effect of presence‐only models is consistent with pseudoabsences not being a “gold standard” when it comes to evaluating SDMs (Carrascal et al., 2015; Jiménez‐Valverde, 2012). Our results highlight that the ability of a model to predict withheld occurrence data is not always a reliable measure of how well it can estimate the true relationship between an environmental gradient and habitat suitability (Warren et al., 2020).

Plots of the abundance–suitability relationship show a characteristic wedge‐shaped structure with regression slopes increasing at higher quantiles, implying lake‐level suitability estimates determine the upper abundance limits of EWM but not the actual observed abundance (Acevedo et al., 2017; VanDerWal, Shoo, Johnson, et al., 2009). From an EWM invasion perspective, it may be inferred that not all lakes that are predicted as highly suitable end up having high abundances, and these differences matter when prioritizing prevention and mitigation actions (see Mikulyuk et al. 2020). This wedge‐shaped relationship is assumed to be the result of an environmental variable restricting the upper limit of abundance, while the precise value remains uncertain because of other covariates that are not typically included in SDMs (e.g., biotic interactions or dispersal constraint; Weber et al., 2017). Moreover, the strength of the wedge‐shaped relationship was greatest for presence–absence models and presence‐only models with proximal pseudoabsence, which have relatively lower discrimination accuracy measures compared to presence‐only models with distant pseudoabsences, which produced both the highest discrimination accuracy values and the smallest difference among quantiles. This decrease in the strength of the wedge‐shaped pattern with increase in model's discrimination accuracy was explained by Jiménez‐Valverde et al. (2021) as the inevitable outcome of information loss (i.e., presence can correspond to multiple abundance values) and stochasticity. In short, these contrasting outcomes reiterate the point that discrimination capacity is a poor indicator of models’ functional accuracy.

## 5 CONCLUSIONS

Like most previous species distribution modeling studies, our study and its findings come with few caveats. For instance, SDMs assume that the species is in equilibrium with its environment (i.e., all available suitable habitats have been invaded), which is especially unlikely for invasive species. Invasive SDMs also assume that the data used for calibration capture the invasive species’ entire range of environmental conditions. While the multidecadal presence of EWM in Minnesota ensures the assumption of equilibrium is less of a problem than it would be for a newer invaded, it still cannot be completely discounted. Hence, future studies on EWM’s distribution and niche might benefit by taking a more exhaustive calibration data that include water temperature measures from EWM’s native range and by developing dynamic models that incorporate EWM’s dispersal potential.

Species distribution models are an increasingly important tool in conservation decision making, and hence, their results and interpretations have tangible consequences. For invasive species, a practical application of SDMs might involve identifying environmental drivers of species distribution and abundance, leveraging this understanding to predict locations of high invasion risk. Here, we identify key ecological drivers of EWM distribution and abundance with a focus on better understanding the invasion processes. Prevention and management of EWM invasion may benefit by taking into consideration these key differences in the drivers of invasion risk versus abundance. Furthermore, through the evaluation of the abundance–suitability relationship, our study highlights the limitations of presence‐only models with pseudoabsences. It is apparent from our results that SDMs designed to maximize discrimination accuracy are not necessarily optimal when it comes to identifying models that accurately predict habitat suitability and species performance. This is especially true when models are based on presence‐only data with pseudoabsences. If presence‐only data are the only available option, random draws of pseudoabsence points are preferred for ensuring that the species–environment relationship is as accurate as possible. More importantly, high‐quality data in the form of systematically collected absence and abundance information are often not available, forcing researchers to rely excessively on presence‐only models. We thus caution against over‐reliance on presence‐only models in species distribution modeling and instead recommend that more resources be allocated to initiating and supporting monitoring programs that collect high‐quality data via systematic monitoring, rather than relying upon opportunistic reporting of presences.

## CONFLICT OF INTEREST

None declared.

## AUTHOR CONTRIBUTIONS

**Shyam M. Thomas:** Conceptualization (lead); Formal analysis (lead); Methodology (equal); Writing‐original draft (lead). **Michael R. Verhoeven:** Data curation (lead); Resources (equal); Writing‐review & editing (equal). **Jake R. Walsh:** Funding acquisition (equal); Writing‐review & editing (equal). **Daniel J. Larkin:** Resources (equal); Supervision (equal); Writing‐review & editing (equal). **Gretchen J. A. Hansen:** Funding acquisition (lead); Methodology (equal); Project administration (lead); Supervision (lead); Writing‐review & editing (equal).

## Supporting information

Table S1‐S2

## Data Availability

All data and R codes associated with this manuscript are available on first author's GitHub account: https://github.com/ShyamThomas under the repository named “Watermilfoil_RF_SDMs.”
