# Supplementary material for: Species distribution models for invasive Eurasian watermilfoil highlight the importance of data quality and limitations of discrimination accuracy metrics
Source: Ecol Evol. 2021 Aug 13;11(18):12567–82. doi: 10.1002/ece3.8002 (PMC8462136; doi:10.1002/ece3.8002)
Supplement: Supplementary file 1 — Table S1‐S2 [file ECE3-11-12567-s001.docx]

**Supplementary Materials**

**Table S1.** ODMAP protocol

| OVERVIEW | |
| --- | --- |
|  | Title: Species distribution models for invasive Eurasian watermilfoil highlight the importance of data quality and limitations of discrimination accuracy metrics |
|  | DOI: TBD |
| Model objective | SDM objective/purpose: Inference, explanation, evaluation, AND comparative assessment across multiple models with different response type/data quality |
|  | Main target output: Continuous measure of invasion risk and frequency of occurrence |
| Taxon | Focal taxa: Eurasian watermilfoil *(Myriophyllum spicatum)* perennial aquatic macrophyte |
| Location | Location of study area: Minnesota, USA |
| Scale of analysis | Spatial Extent (Lon / Lat): -97.2 to -89.4 E, 43.49 to 49.4 N |
|  | Spatial resolution: individual lake-level predictions |
|  | Temporal extent/time period: Data collected 1995-2019 |
|  | Temporal resolution: Composite time slice |
|  | Type of extent boundary (e.g., rectangular, natural, political): State political boundary |
| Biodiversity data overview | Observation type: standardized point-intercept data from lake surveys |
|  | Response/data type: presence-absence, presence-only, and abundance (frequency of occurrence) |
| Type of predictors | 11 Lake-level predictors of lake morphometrics, water temperature, water chemistry and physical attributes, surrounding land-use (road density & stream connectivity) |
| Conceptual model | Test of data quality and evaluation metrics: Descriptive/comparative assessment using different species response data that results in presence-absence, presence-only, and abundance based SDMs; evaluation of discrimination accuracy across different models by leveraging abundance measures |
| Assumptions | Assumes that different response data is likely to result in notable differences in both predictions and importance of key habitat suitability drivers, and that the fine scale lake-specific predictors are likely capture these differences. Also assumes conventional discrimination metrics like AUC may not capture the true nature of species-environment relationship for occurrence models, and the species-environment relationship may be better captured through functional accuracy measures such as relationship between predicted suitability and abundance. |
| SDM algorithms | Model algorithms: Random forests |
|  | Justification of model complexity: Random forest models are nonparametric models that capture nonlinear interactions between predictors and response variables, do not assume normal distributions, and provide a direct assessment of predictor variable importance. All of which allows effective comparison across models. |
|  | Model averaging/ensemble modelling: Results averaged across trees in the boosted regression tree framework. Model evaluation metrics averaged across five validation folds. |
| Model workflow | Models for all species used the same set of minimally correlated predictor variables, fitting boosted regression trees to presence and absence observations within a threshold distance from known presences. For each species, the threshold was determined by optimizing net AUPRC across a range of possible distances, using five-fold cross-validation. Models were evaluated on a suite of metrics within the optimum threshold training area, again by five-fold cross-validation. Modeled probabilities of presence were then projected for each species within the training area (higher confidence) and across all Level III ecoregions where the species was observed (lower confidence outside training area.) |
| Software, codes and data | Software: R version 3.6.2, packages ‘sdm’, 'randomForest'; 'raster', 'sf', ‘sp’, ‘blockCV’, and others such as ‘tidyverse’ for data processing |
|  | All codes and scripts, and datasets are archived as repositories in first author’s GitHub account |
|  | Data downloaded from multiple sources: <https://www.dnr.state.mn.us/invasives/ais/infested.htm> (EWM invasion data)  <https://www.pca.state.mn.us/water/water-quality-data> (MPCA water quality data)  <https://gisdata.mn.gov/organization/us-mn-state-dnr> (lake morphometric data)  <https://lagoslakes.org/> (landscape data) |
| DATA | |
| Biodiversity data | Taxon name: Eurasian watermilfoil (*Myriophyllum spicatum L.)* |
|  | Taxonomic reference system: followed USDA Plants Database (2020) |
|  | Ecological level: species |
|  | Sampling design: Compilation of systematic point-intercept surveys, points cover lake littoral area |
|  | Sample size: >1500 lake surveys in total; 365 EWM infested lakes |
|  | Scaling: All response and predictor data are collected at at the individual lake scale |
|  | Data cleaning/filtering: Removed all lakes that don’t have full information on any of the 11 lake predictors |
|  | Absence data: Point intercept surveys recorded all species present without taxon preference; hence absences were implied for any focal taxa. We also included pseudoabsence data from different spatial draws of lakes that were not part of the survey (see Methods for details). |
|  | Selection of training data: Multiple approaches were used; fivefold cross-validation, and single-fold cross-validation by leaving out 30 percent as test data, and spatially blocked cross-validation. |
|  | Selection of validation data: All presences and absences in withheld testing fold  Test data: Abundance measures were used as independent test data to capture functional accuracy |
| Predictor variables | Predictor variables: See Table 1 in text |
|  | Data processing and scaling: Fire polygons and road networks were rasterized at resolution of DEM, and all environmental predictor variables were resampled to the resolution of the DEM by bilinear interpolation |
|  | Dimension reduction: We calculated pairwise correlations between possible predictor variables. Only variables had Pearson’s correlations of rho < 0.7 were included. |
|  | Data sources: same as predictor variables |
|  | Spatial extent: State of Minnesota  Temporal extent: Between 1990-2018 |
| MODEL | |
| Model settings & complexity | Models settings: All occurrence models – sdm function in sdm package with default settings and preselected randomForest algorithm, and ntree = 500.  All abundance models – separate models using randomForest alogirthm, and ntree = 500 |
| Model estimates /  Model selection | Assessment of variable importance: Relative reduction in model discrimination statistic AUC, and mean square error based on permutation of each variable, averaged across all trees in the final model, as implemented by default in the “sdm” and “randomForest” package |
| Model estimates /  Model selection | Model selection strategy: the random forest method does not require creation of parsimonious models or estimation of coefficients. Instead, we ensured multiple presence-absence, presence-only and abundance models were evaluated using same predictors to gain a thorough comparative assessment. |
| Model averaging / Ensembles | Method for model averaging random forest method inherently integrates over all the fitted trees in the model. |
| Non-independence correction | Spatial autocorrelation in residuals: we included models with a spatial autocovariate |
| Threshold selection | Threshold selection: We report continuous probabilities across all models. For occurrence models based on presence-absence and presence-only data, we used threshold independent AUC statistic, as well as threshold-dependent TSS and Kappa statistics. For TSS AND Kappa statistics, threshold value was selected when sensitivity matched specificity. |
| ASSESSMENT | |
| Performance statistics | Performance statistics estimated on training data/validation data: For occurrence-based models – AUC, TSS, & Kappa statistics. For abundance (frequency of occurrence) models – mean squared error statistic. |
|  | Performance statistics estimated on test data: Only for occurrence models, we had a truly independent test data – frequency of occurrence. Correlation between predicted suitability and frequency off occurrence was used as a functional accuracy statistic. |
| Plausibility check | Response plots: Partial effects plots to visualize relationships between key predictors and predicted probability of invasion risk and occurrence frequency based on models that withheld occurrence and abundance observations |
| PREDICTION (no predictions or maps were made outside of training data, hence NA) | |

**Table S2.** Coefficients of quantile regression models at the 50^th^ (***𝛕_50th_***), 75^th^ (***𝛕_75th_***), and 90^th^ (***𝛕_90th_***) percentile levels. Difference between 90^th^ and 50^th^ quantile regression coefficients (D) is the measure of the strength of the wedge-shaped relationship.

|  | ***𝛕_50th_*** | ***𝛕 _75th_*** | ***𝛕 _90th_*** | | ***D*** (𝛕 _90th_-𝛕 _50th_) |
| --- | --- | --- | --- | --- | --- |
| **Presence-absence** | 0.038 (0.015-0.054) | 0.18 (0.16-0.23) | | 0.40 (0.34-0.46) | 0.362 |
| **Presence-absence + Autocovariate** | 0.039 (0.02-0.06) | 0.17 (0.14-0.21) | | 0.39 (0.33-0.43) | 0.361 |
| **Presence-only (random pseudoabsence)** | 0.00 (0.00-0.02) | 0.10 (0.07-0.16) | | 0.34 (0.27-0.36) | 0.34 |
| **Presence-only (random pseudoabsence) + Autocovariate** | 0.00 (0.00-0.02) | 0.13 (0.10-0.16) | | 0.33 (0.29-0.41) | 0.33 |
| **Presence-only (distant pseudoabsence)** | 0.00 (0.00-0.00) | 0.06 (0.056-0.09) | | 0.25 (0.22-0.31) | 0.25 |
| **Presence-only (distant pseudoabsence)**  **+ Autocovariate** | 0.00 (0.00-0.00) | 0.03 (0.02-0.04) | | 0.20 (0.17-0.21) | 0.20 |
| **Presence-only (proximal pseudoabsence)** | 0.00 (0.00-0.02) | 0.14 (0.10-0.21) | | 0.41 (0.35-0.43) | 0.41 |
| **Presence-only (proximal pseudoabsence)**  **+ Autocovariate** | 0.00 (0.00-0.012) | 0.14 (0.11-0.19) | | 0.39 (0.34-0.41) | 0.39 |
